# Effect of undissolved Nb carbides on mechanical properties of hydrogen-precharged tempered martensitic steel

**DOI:** 10.1038/s41598-020-68653-4

**Published:** 2020-07-16

**Authors:** Hyun Joo Seo, Jang Woong Jo, Jae Nam Kim, Kitae Kwon, Junmo Lee, Sangwoo Choi, Taekyung Lee, Chong Soo Lee

**Affiliations:** 1 Graduate Institute of Ferrous Technology, Pohang University of Science and Technology (POSTECH), Pohang, 37673 South Korea; 2 Pohang Research Lab, POSCO, Pohang, 37877 South Korea; 3 School of Mechanical Engineering, Pusan National University, Busan, 46241 South Korea

**Keywords:** Mechanical properties, Metals and alloys

## Abstract

Nb carbides have attracted significant attention to enhance the resistance of tempered martensitic (TM) steel to hydrogen embrittlement (HE). However, previous studies have elucidated the role of Nb carbides in HE resistance without categorizing their types (i.e., undissolved and newly precipitated). This study focuses on the effect of “undissolved” Nb carbides on the tensile and fatigue properties of hydrogen-precharged TM steels. It validated the following two factors for the HE resistance of the TM steels containing undissolved Nb carbides: hydrogen-trapping by the carbides and refinement of prior austenite grain. The former factor rarely affected the HE resistance owing to the interfacial incoherency between the undissolved carbides and ferritic matrix. Such results are distinguished from previous studies focusing on the newly precipitated carbides. In contrast, the latter factor contributed significantly to the HE resistance via the decrease in hydrogen contents per unit surface of prior austenite grain boundaries.

## Introduction

Hydrogen embrittlement (HE) indicates the deterioration in mechanical properties owing to hydrogen atoms inside a ferrous alloy^1,2^. HE is particularly important for the use of high-strength steels, such as a tempered martensitic (TM) steel. The TM structure can yield a high strength that exceeds 1.2 GPa after a simple heat treatment, rendering this steel important for various industries. Nevertheless, a high density of defects in the TM structure renders it highly vulnerable to HE^3^. Consequently, this has resulted in increasing demands for an enhanced HE resistance of TM steels.

The addition of Nb is an effective approach to increase the HE resistance of TM steels. It has been reported that HE resistance increases owing to the hydrogen trapping by Nb carbides and the refinement of prior austenite grains (PAGs). Such factors hindered the diffusion and concentration of the hydrogen present inside materials^4^. A recent study^5^ suggested a different mechanism for achieving an improvement in HE resistance, wherein Nb addition decreased the Σ3 boundary fraction in lath martensites. However, previous studies have primarily investigated Nb carbides, which are newly precipitated during a tempering process. The other type of Nb carbides (i.e., “undissolved” carbides) has attracted significantly less attention despite the fact that their resultant amounts are not negligibly small.

Therefore, the effect of undissolved Nb carbides on the HE resistance of TM steels was investigated in this study. Four hydrogen-precharged TM steels were evaluated under uniaxial and cyclic loading conditions by considering the application environment of the material.

## Material and methods

In this study, two types of TM steels were used separately, i.e., those with the chemical compositions of Fe–0.5C–0.5Si–0.7Mn–1.0Cr–0.8Mo and Fe–0.5C–0.5Si–0.7Mn–1.0Cr–0.8Mo–0.02Nb (numbers in mass percentage). These steels were homogenized at 1,473 K for 2 h, followed by two different thermomechanical processes. The sections of steels were hot-rolled, austenitized at 1,193 K for 1 h, quenched to oil bath at 328 K, and then tempered at 823 K for 1 h. They are denoted as Nb0-A and Nb2-A, respectively, where the numbers represent their Nb compositions. Other sections of the steels were subjected to a similar thermomechanical process, except for a different austenitizing condition (1,253 K for 4 h) to induce a similar PAG size. They are denoted as Nb0-B and Nb2-B, respectively.

Sections of TM steel samples were electrochemically polished in a solution of 95% acetic acid and 5% perchloric acid for electron backscatter diffraction (EBSD) analysis. Other sections were chemically etched in a 4% nital solution for scanning electron microscopy (SEM). Nb2-A steel was subjected to a carbon extraction replica process and then observed through transmission electron microscopy (TEM), which was integrated with energy-dispersive spectroscopy (EDS).

The HE resistance was evaluated via a slow strain rate test (SSRT) and a high-cycle fatigue (HCF) test after hydrogen precharging. Sections of SSRT samples were hydrogen-precharged in 0.1-M NaOH solution for 48 h at a current density of 20 A m^−2^, whereas others remained free from hydrogen. The SSRT was conducted at a strain rate of 10^−5^ s^−1^ using cylindrical specimens, which were measured to have a gage diameter and gage length of 4 and 25 mm, respectively. The tests were repeated three times to confirm the data reproducibility. Sections of HCF samples were hydrogen-precharged in a solution of 3% NaCl and 0.3% NH_4_SCN for 48 h at a current density of 10 A m^−2^. The HCF test was performed using cylindrical specimens that exhibited a gage diameter and gage length of 5 and 10 mm, respectively. The applied stress ratio and frequency were 0.1 and 10 Hz, respectively. Subsequently, these specimens were coated with Zn to prevent the hydrogen release during the test. Thermal desorption analysis (TDA) was introduced to measure the amount of internal hydrogen in the investigated TM steels.

## Results and discussion

**Figure 1:**
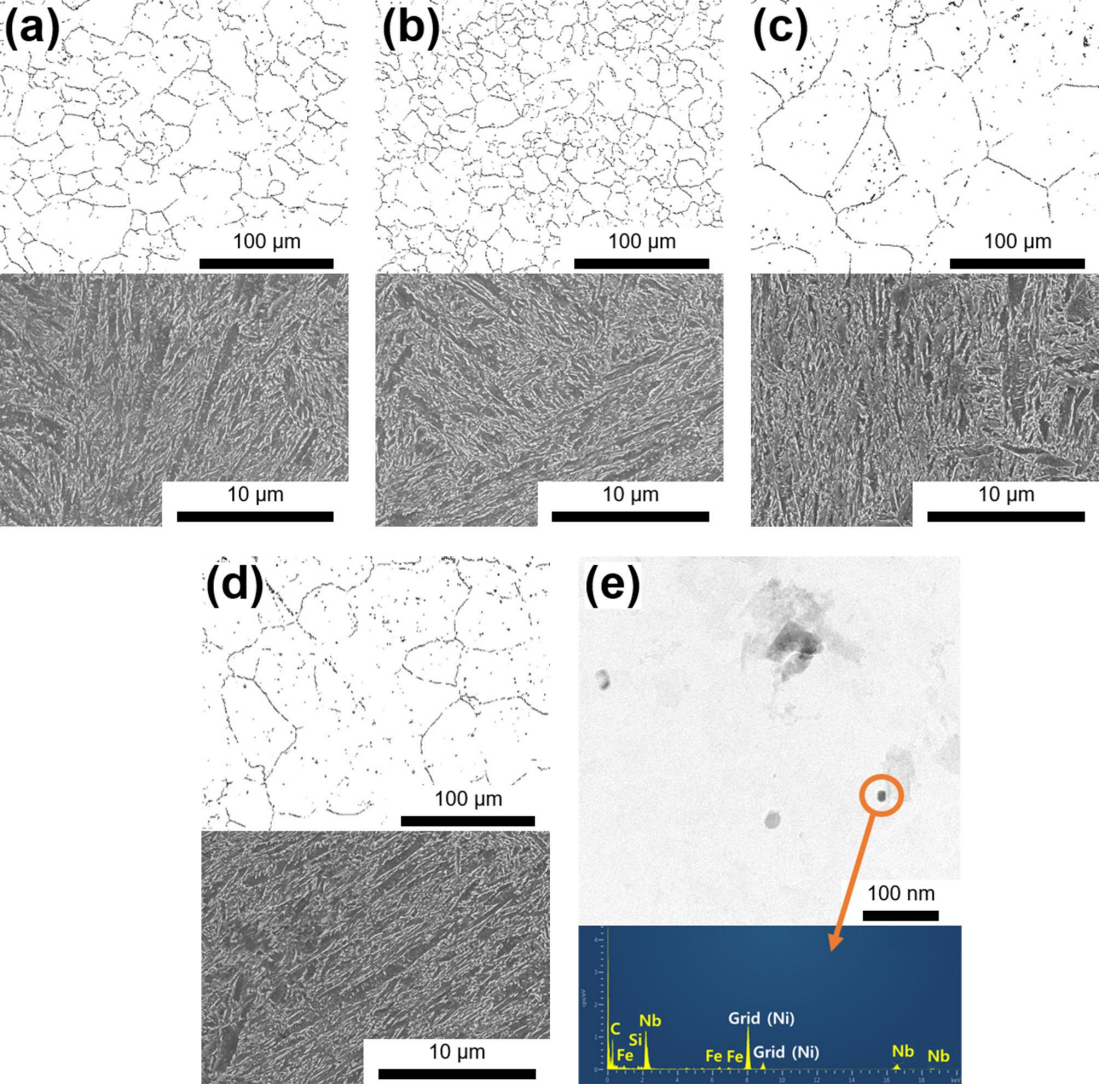
Microstructure of the investigated TM steels: EBSD and SEM micrographs of (**a**) Nb0-A, (**b**) Nb2-A, (**c**) Nb0-B, and (**d**) Nb2-B; TEM micrograph and corresponding EDS analysis of Nb carbides in (**e**) Nb2-A. “*d*_PAG_” indicates an average diameter of PAGs.

The investigated steels demonstrated a typical TM structure with different microstructural characteristics that were governed by the Nb composition and austenitizing conditions (Fig. 1). The PAG was determined using the EBSD analysis based on the grain-boundary misorientation angle from 20° to 47°^6^. The PAG size of the Nb2-A steel (*d*_PAG_ = 13.1 μm) was half of that of the Nb0-A steel (*d*_PAG_ = 25.6 μm), suggesting a significant PAG refinement by Nb addition. In contrast, Nb0-B and Nb2-B steels possessed similarly coarse PAGs as intended (*d*_PAG_ = 44.2 μm and 49.2 μm, respectively) due to the excessive austenitizing temperature and duration.

TEM–EDS analysis revealed the distribution of nanoscale (13 nm in average) Nb carbides in Nb2-A steel. These carbides are distinct from those newly precipitated during the tempering process for two reasons. First, the thermomechanical processing route employed in this work did not allow a complete dissolution of Nb carbides. This deduction was confirmed by the dissolution temperature (1,425 K) calculated from the ThermoCalc software with TCS Steel and Fe Alloys Database ver. 7^7^. Nb carbides were precipitated during the hot rolling at the temperature lower than 1,425 K. Sections of these carbides were not dissolved during the subsequent austenitizing step at 1,193 K, thereby remaining in the final microstructure. Second, the newly precipitated carbides demonstrate a needle or plate shape with the Baker-Nutting orientation relationship with a ferritic matrix^8^, which did not match the round shape of the Nb carbides shown in Nb2-A steel.

The precipitation during the hot rolling assisted in limiting a size of undissolved carbides to nanoscale, because strain-induced dislocations acted as a nucleation site^9^. Such fine carbides induced the pinning effect and the resultant PAG refinement in Nb2-A steel^10^. Meanwhile, the ThermoCalc simulation also calculated the volume fraction of Nb carbides at each austenitizing temperature: 0.022% at 1,193 K (Nb2-A) and 0.020% at 1,253 K (Nb2-B). The significantly low difference (0.002%) between the two conditions suggests the negligible influence of austenitizing temperature on the fraction of carbides in this work.

**Figure 2:**
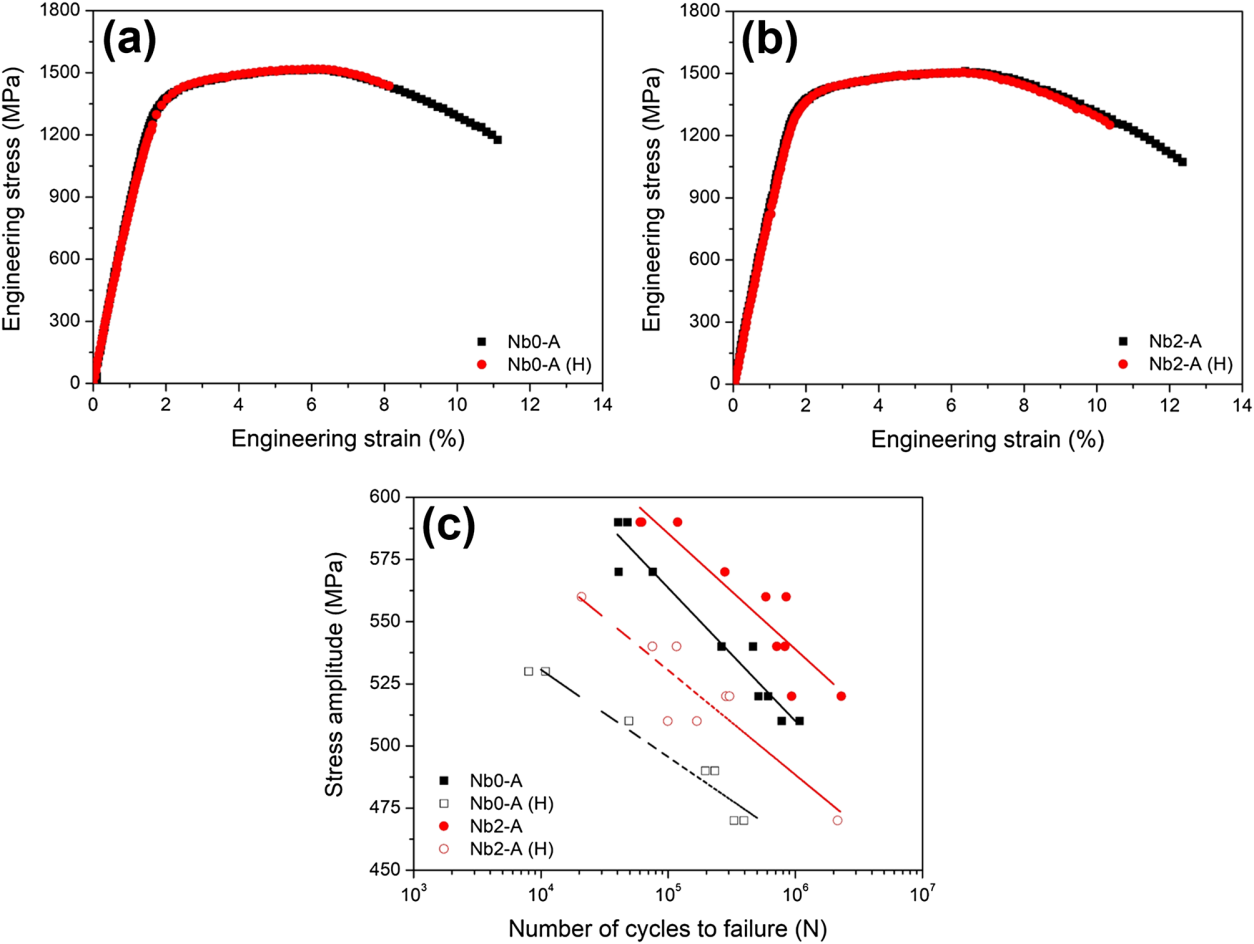
Mechanical properties of Nb0-A and Nb2-A steels before and after hydrogen precharging: (**a**,**b**) SSRT and (**c**) HCF. The symbol “(H)” indicates the hydrogen precharging before the test.

Hydrogen effects were investigated for the Nb0-A and Nb2-A steels in uniaxial and cyclic deformation modes (Fig. 2). These steels possessed similar tensile properties when they were free from hydrogen. However, hydrogen precharging resulted in degraded tensile properties in different ways. Nb0-A steel exhibited a larger loss of elongation (23.8%) as compared to that of Nb2-A steel (18.3%). Regarding the fatigue properties, Nb2-A steel showed a higher fatigue resistance than its counterpart even prior to the hydrogen precharging. This is attributed to the fine PAG structure that interrupts fatigue crack propagation^11^. The superiority of Nb2-A steel remained after both samples were subjected to the hydrogen precharging. Combining the results from the SSRT and the HCF test, it is concluded that Nb addition to TM steel enhanced HE resistance in both uniaxial and cyclic deformation modes.

**Figure 3:**
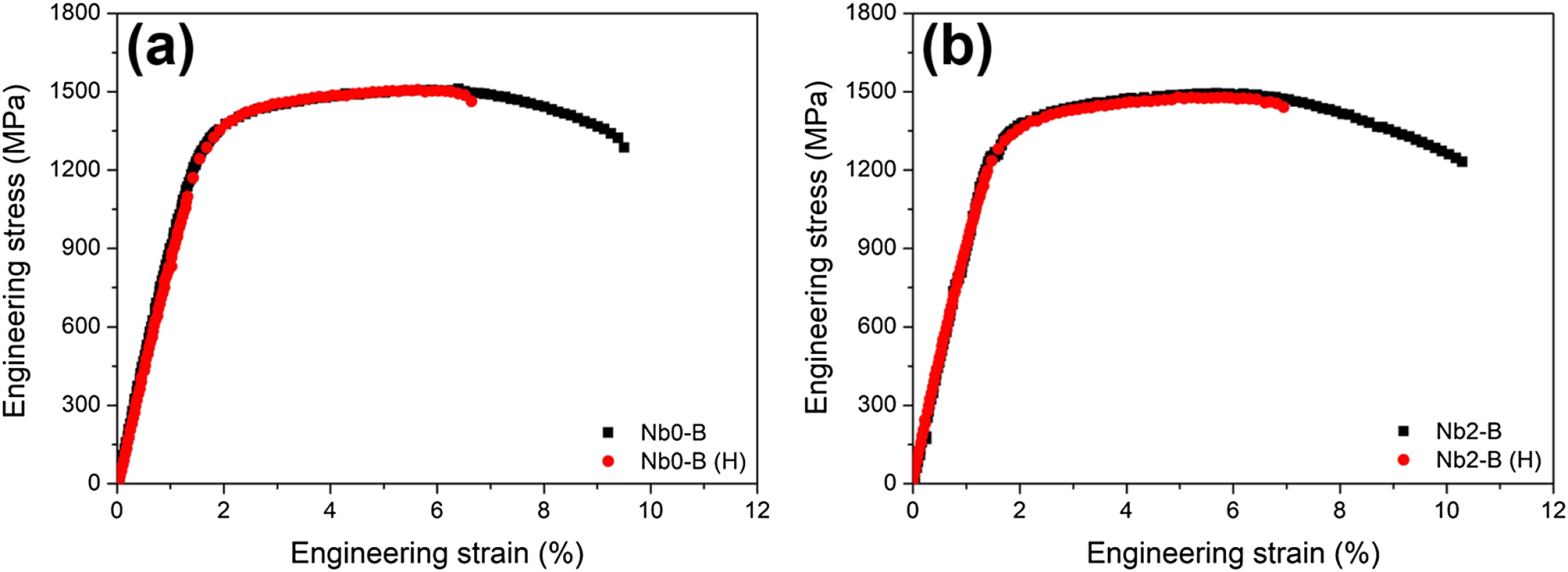
SSRT stress–strain curves of (**a**) Nb0-B and (**b**) Nb2-B steels. The symbol “(H)” indicates hydrogen-precharging before the test.

In general, the HE resistance of steel is highly dependent on its ultimate tensile strength (UTS)^12^. The present TM steels were tailored to reflect similar UTS values of ~ 1.5 GPa, which excludes the UTS factor from discussions hereafter. Besides the UTS factor, there are two possible mechanisms that Nb carbides enhance HE resistance of Nb2-A steel: hydrogen trapping by PAG boundaries (PAGBs) or by carbide-matrix interfaces. The pinning effect induced by Nb carbides may suppress a grain growth, thereby inhibiting a decrease in hydrogen-trapping sites (i.e., PAGBs). Otherwise, Nb carbides may act as a hydrogen-trapping site by themselves. The SSRT results of Nb0-B and Nb2-B steels clarified which mechanism was activated in the present case (Fig. 3). Nb0-B and Nb2-B steels demonstrated similar losses of elongation (29.1% and 28.6%, respectively) in contrast to Nb0-A and Nb2-A steels. In addition, as shown in Fig. 1, these steels have the similar size of PAGs despite their different Nb compositions. These results indicate the negligible effect of the Nb composition on HE resistance under the condition of similar PAG size. Consequently, PAG size is more crucial factor in HE resistance of the investigated steels.

**Figure 4:**
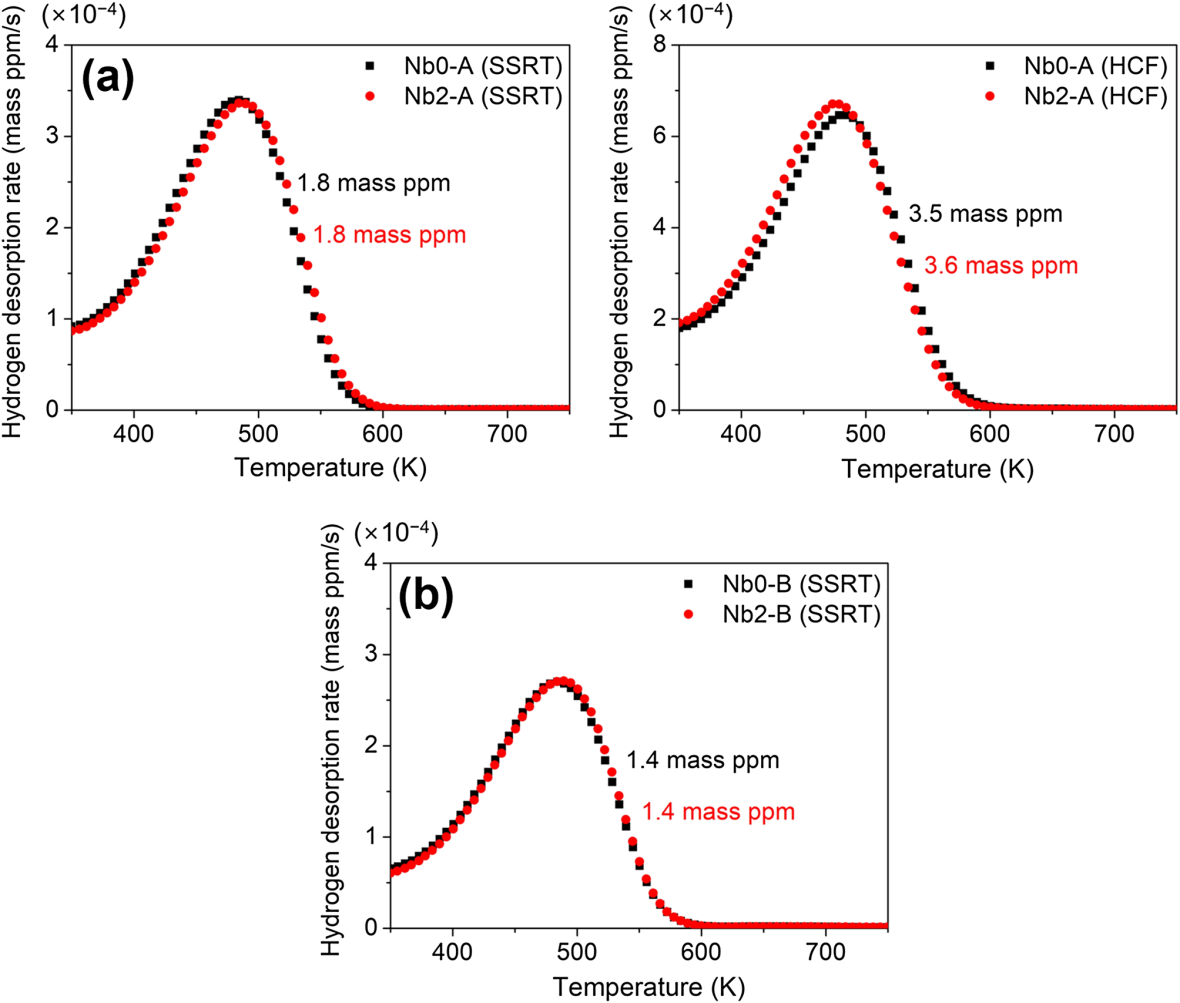
Hydrogen desorption rate as a function of temperatures for (**a**) Nb0-A and Nb2-A steels and (**b**) Nb0-B and Nb2-B steels.

For further discussion, the amount of internal hydrogen was studied through TDA for the investigated TM steels (Fig. 4). Despite the different Nb compositions, Nb0-A and Nb2-A steels presented similar amounts of internal hydrogen: 1.8 mass ppm for the SSRT specimens and 3.5 mass ppm for the HCF specimens. Nb0-B and Nb2-B steels also showed the similar hydrogen contents: 1.4 mass ppm for the SSRT specimens. These results suggest that the Nb addition hardly contributed to the total amount of hydrogen trapping for the investigated steels. This is in good accord with previous studies^3,5^. The primary hydrogen-trapping sites in TM steel are high-angle boundaries of PAG, packets, blocks, and laths as well as precipitates^13,14^. According to a recent study of Zhang et al*.*^5^, the Nb addition to a martensitic steel rarely changes the total amount of hydrogen trapped by these boundaries, although the local hydrogen concentration significantly varied depending on the boundary type. Kim et al*.*^3^ also concluded the negligible variation in the total hydrogen concentration in coarse PAG microstructure (i.e., *d*_PAG_ ≥ 10 μm); note that the present TM steels also possessed the similar microstructure.

Based on the TDA results, it is not the total amount of hydrogen trapping but the local hydrogen concentration that induced the improved HE resistance of Nb2-A steel. A slow-rate plastic deformation (e.g., SSRT) causes the local hydrogen concentration at PAGBs, which act as a crack initiation site with further straining^15^. The PAG refinement in Nb2-A steel resulted in an increasing fraction of PAGBs. Considering the similar hydrogen contents in Nb0-A and Nb2-A steels, the increasing PAGB fraction gave rise to the reduction of hydrogen concentration per unit surface of boundaries^16^. Such an alleviated hydrogen concentration delayed the HE fracture of Nb2-A steel. In contrast, Nb0-B and Nb2-B steels exhibited the comparable HE resistance, because the excessive austenitizing process gave rise to the similar PAG sizes regardless of the Nb compositions.

The negligible effect of Nb compositions shown in Nb0-B and Nb2-B steels may appear inconsistent with previous studies, which have considered a hydrogen trapping by Nb carbides as the primary factor for HE resistance of TM steels^14,17^. The hydrogen trapping by Nb carbides has been experimentally proven by various methods, such as TDA^18^ and small-angle neutron scattering^19^. It should be noted, however, such an interpretation is only applicable to the newly precipitated Nb carbides with a high interfacial coherency. Hydrogen-trapping capability is highly dependent on the interfacial coherency between the carbide and matrix^20^. It is worth noting that the Nb carbides in the present TM steels were undissolved during the austenitizing process. These carbides are incoherent with the ferritic matrix and hence unable to trap internal hydrogen in the hydrogen environment^21^. Therefore, the traditional mechanism of Nb carbides is inapplicable to TM steels possessing a high portion of undissolved carbides. The improved HE resistance in such materials is interpreted in light of the reduced hydrogen concentration due to the increasing PAGB fraction, as suggested above. This alternative mechanism provides a reasonable explanation for the improved HE resistance of Nb2-A steel with undissolved Nb carbides and PAG refinement.

## Conclusions

Herein, the effect of undissolved Nb carbides on the HE resistance of TM steel was investigated in uniaxial and cyclic deformation modes. The Nb addition resulted in undissolved Nb carbides and PAG refinement. Nb2-A steel containing 0.02 mass percentage of Nb exhibited an improved HE resistance in both deformation modes. However, Nb2-B steel showed an HE resistance that was similar to that of Nb0-B steel. The results suggested the significance of PAG refinement in enhancing the HE resistance of the TM steel that comprises undissolved Nb carbides. The PAG refinement increased the PAGB fraction, thereby alleviating the local hydrogen concentration. Hydrogen trapping by undissolved Nb carbides rarely contributed to the increasing HE resistance owing to the interfacial incoherency with the ferritic matrix.
